# Templated Bipolar Host Materials for Blue Phosphorescent Organic Light-Emitting Devices with Negligible Efficiency Roll-Offs

**DOI:** 10.3390/molecules31010012

**Published:** 2025-12-19

**Authors:** Hong Huang, Tao Hua, Nengquan Li, Youming Zhang, Manli Huang, Xiaolu Zhou, Shaoqing Zhuang, Guohua Xie

**Affiliations:** 1 Institute of Technology for Future Industry, School of Science and Technology Instrument Application Engineering, Shenzhen University of Information Technology, Shenzhen 518172, China; 2 Wuhan Sunshine Optoelectronics Tech Co., Ltd., New Energy Building, No. 999 Gaoxin Avenue, Wuhan 430074, China; 3 Institute of Flexible Electronics (IFE, Future Technologies), Institute of Future Display Technology, Tan Kah Kee Innovation Laboratory, Xiamen University, Xiamen 361102, China

**Keywords:** CBP derivatives, phosphorescent organic light-emitting diodes, host materials, efficiency roll-offs

## Abstract

Host engineering is one of the most efficient approaches to maximizing the electroluminescent performance of organic light-emitting devices. Herein, two carbazole-based N,N′-Dicarbazolyl-4,4′-biphenyl (CBP) derivatives, (9-(4′-(9H-carbazol-9-yl)-[1,1′-biphenyl]-4-yl)-3-(3-(1-phenyl-1H-benzo[d]imidazol-2-yl)phenyl)-9H-carbazole (**CBPmBI**), and (9-(4′-(9H-carbazol-9-yl)-[1,1′-biphenyl]-4-yl)-9H-carbazol-3-yl)diphenylphosphine oxide (**CBPPO**), were designed as bipolar hosts for blue phosphorescent devices. By introducing the electron-withdrawing groups to the backbone of CBP, the bipolar hosts exhibited high triplet energy, enhanced thermal stability, and balanced charge transport. The device constructed with the blue guest emitter bis[2-(4,6-difluorophenyl) pyridinato-C2,N]iridium (III) (FIrpic) showed the excellent electroluminescence performance. For instance, the **CBPPO**-based devices achieved a maximum current efficiency of 28.0 cd/A, a power efficiency of 25.8 lm/W, and an external quantum efficiency of 14.4%. Notably, the external quantum efficiency retained at14.1% under the brightness of 5000 cd/m^2^, featuring the negligible efficiency roll-off.

## 1. Introduction

Organic light-emitting devices (OLEDs) dominate modern display and solid-state lighting technologies due to their flexibility, low power consumption, and high color purity [1,2,3,4,5,6,7,8]. Among them, blue phosphorescent OLEDs (PhOLEDs) are indispensable for full-color displays and white lighting systems. However, their performance remains inferior to red and green PhOLEDs—this bottleneck is primarily attributed to the lack of ideal host materials that simultaneously possess sufficient triplet energy (E_T_) to confine excitons on blue emitters and balanced charge-transporting capabilities to avoid high driving voltages [9,10,11,12].

N,N′-Dicarbazolyl-4,4′-biphenyl (CBP), a carbazole-based compound, has long served as a benchmark host for green and red PhOLEDs [13,14]. Its popularity stems from good hole-transporting properties (derived from electron-donating carbazole units) and high solubility in common organic solvents, which simplifies material purification and device fabrication. Nevertheless, CBP is unsuitable for blue PhOLEDs: its relatively low E_T_ (2.56 eV) is lower than that of the widely used blue phosphorescent dopant FIrpic (E_T_ = 2.65 eV) [12]. This energy mismatch triggers reverse energy transfer from FIrpic to CBP, resulting in severe exciton loss and low device efficiency.

To address CBP’s limitations, researchers have developed two main modification strategies, both with inherent drawbacks. The first adjusts CBP’s molecular backbone to elevate E_T_—for example, N,N′-dicarbazolyl-3,5-benzene (mCP), synthesized by replacing CBP’s biphenyl unit with a single benzene ring, achieves an E_T_ of 2.9 eV [15] but suffers from reduced thermal stability (low glass transition temperature, T_g_) due to lower molecular weight, leading to film recrystallization or host-emitter phase separation during fabrication/operation [16,17]. The second strategy incorporates steric groups or non-conjugated linkages (e.g., 4,4′-bis(9-carbazolyl)-2,2′-dimethylbiphenyl, CDBP and bis(4-(9-carbazolyl)phenyl)diphenylsilane, CPSiCBP; E_T_ = 3.0 eV [18,19,20,21,22,23,24,25,26]) to disrupt conjugation, CDBP introduces two methyl groups at the 2- and 2′-positions of the biphenyl unit to create steric hindrance, while CPSiCBP uses a tetraphenylsilane group as a non-conjugated linkage between the two carbazole-containing segments. Although these materials have sufficient triplet energy to host FIrpic, their hole-transporting properties are significantly compromised by the steric or non-conjugated modifications. This poor electron transport leads to high driving voltages in devices, as more energy is required to inject and transport electrons from the electron-transporting layer to the emissive layer. High driving voltages not only increase power consumption but also accelerate material degradation (due to increased Joule heating and charge accumulation at the interfaces), shortening the operational lifetime of practical devices [20,21,22,23,24,25,26].

Herein, we propose a facile solution: introducing electron-withdrawing groups (EWGs) to the CBP backbone to simultaneously resolve the above contradictions. This work synthesized two CBP derivatives (**CBPmBI** and **CBPPO**) via efficient coupling reactions. The derivatives exhibit high E_T_ (2.67 eV, exceeding Firpic’s), enhanced thermal stability (T_g_ = 147–157 °C, decomposition temperature T_d_ = 460–494 °C), and balanced charge transport. Blue PhOLEDs with **CBPPO** as the host achieve optimal performance (maximum external quantum efficiency EQE_max_ = 14.4%, current efficiency η_c,max_ = 28.0 cd/A) and negligible efficiency roll-off (14.1% EQE at 5000 cd/m^2^). This study highlights a simple EWG-modification strategy for tuning CBP derivatives, offering a promising route to high-performance blue PhOLED hosts.

## 2. Results and Discussion

All relevant information regarding the Materials and Measurements section is available in the Supplementary Information [27,28,29,30].

### 2.1. Synthesis and Characterization

**Scheme 1:**
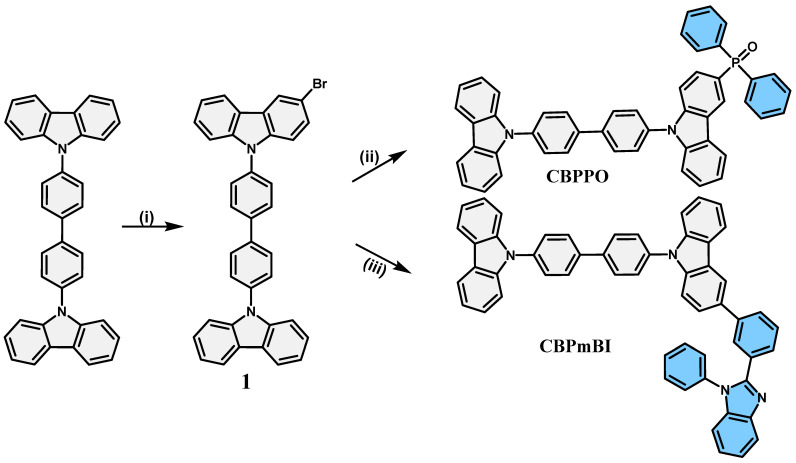
The synthesis routes of the compounds **CBPPO** and **CBPmBI**. Reagents and condition: (i) NBS, CHCl_3_, Silicon, rt, 48 h; (ii) Zn, NiCl_2_∙6H_2_O, 2,2′-bipyridine, DMAc, diphenylphosphine oxide, 110 °C, 24 h; (iii) Pd(PPh_3_)_4_, Toluene, K_2_CO_3_(2M), Ethanol, 1-phenyl-2-(3-(4,4,5,5-tetramethyl-1,3,2-dioxaborolan-2-yl)phenyl)-1H-benzo[d]imidazole, 100 °C, 24 h.

The novel bipolar host materials (**CBPPO** and **CBPmBI**) were prepared following the synthetic pathway outlined in Scheme 1. Using commercially accessible CBP as the starting material, the key intermediate—9-(4′-(9H-carbazol-9-yl)-[1,1′-biphenyl]-4-yl)-3-bromo-9H-carbazole (compound **1**)—was synthesized via bromination of **CBP** using N-bromosuccinimide (NBS). This bromination reaction yielded compound **1** in a high yield of 95%, by adopting the protocol documented in the literature [31]. The final product **CBPPO** was obtained through a one-pot Ni (II)/Zn-catalyzed cross-coupling reaction [32] between diphenylphosphine oxide and the bromide- activated CBP derivative (compound **1**) at 100 °C, yielding a good product output. Notably, this reaction did not require low-temperature conditions, and the crude product could be easily purified via silica gel column chromatography. This synthetic approach thus offers distinct advantages compared to conventional coupling reactions involving n-butyllithium (n-BuLi), chlorodiphenylphosphine, or hydrogen peroxide (H_2_O_2_), which are often more complex and demanding.

In contrast, the other target host material **CBPmBI** was synthesized via a Suzuki coupling reaction. Specifically, this reaction was conducted between compound **1** and 1-phenyl-2-(3-(4,4,5,5-tetramethyl-1,3,2-dioxaborolan-2-yl)phenyl)-1H-benzo[d]imidazole, leading to the formation of **CBPmBI**. Detailed synthetic procedures and comprehensive characterization data for these new compounds are provided in the Supporting Information. Both are white powders, highly soluble in THF, CH_2_Cl_2_, and toluene. The target compounds underwent additional purification via recurrent temperature-gradient vacuum sublimation (critical for device performance). Their chemical structures were verified through APCI-MS, ^1^H NMR, and ^13^C NMR (Figures S1–S4).

### 2.2. Thermal Properties

**Figure 1:**
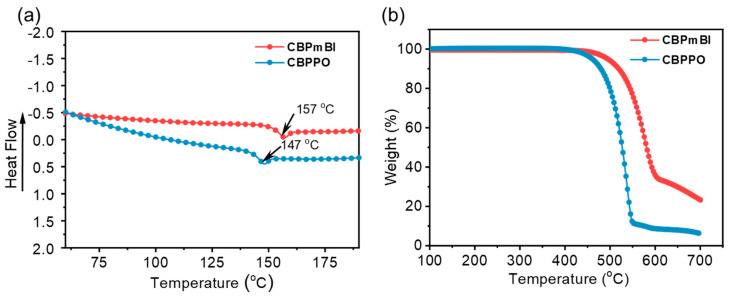
(**a**) DSC curves of **CBPPO** and **CBPmBI**. (**b**) TGA curves of **CBPPO** and **CBPmBI**.

Thermal performance of CBP derivatives (**CBPPO**, **CBPmBI**) was assessed using thermogravimetric analysis (TGA) and differential scanning calorimetry (DSC) under N_2_ atmosphere (to eliminate O_2_/moisture interference for accurate results). Both show excellent thermal stability (critical for organic optoelectronics, as poor stability causes film degradation/volatilization during device fabrication/operation; Figure 1).

**CBPPO** has a decomposition temperature (T_d_) of 460 °C, while **CBPmBI** reaches 494 °C. These values are far higher than the typical processing temperatures for OLEDs, where vacuum thermal evaporation generally stays below 400 °C. As a result, both materials can endure the thermal stress during device fabrication without suffering obvious weight loss or structural decomposition. The glass transition temperatures (T_g_) of **CBPPO** and **CBPmBI** are 147 °C and 157 °C, respectively, which are notably higher than the 63 °C T_g_ of pristine CBP [33]. The main reason for this T_g_ improvement lies in the electron-withdrawing groups (EWGs) introduced to the CBP molecular backbone. These groups disrupt CBP’s original symmetric structure, creating an asymmetric molecular configuration. This asymmetry enhances intermolecular entanglement in the amorphous films, which boosts the materials’ resistance to plastic deformation and thus elevates their T_g_ values. It also suppresses recrystallization, a phenomenon that would cause phase separation between the host and emitter materials, further leading to reduced efficiency and shortened lifetime of OLEDs.

High T_g_/T_d_ is valuable for OLEDs: it prevents emissive layer phase separation under heat (vacuum deposition/high-brightness operation) and ensures long-term film morphological stability for consistent performance. Notably, these values also enable compatibility with vacuum evaporation, facilitating fabrication of uniform, high-quality thin films for high-performance devices.

### 2.3. Photophysical Properties

**Figure 2:**
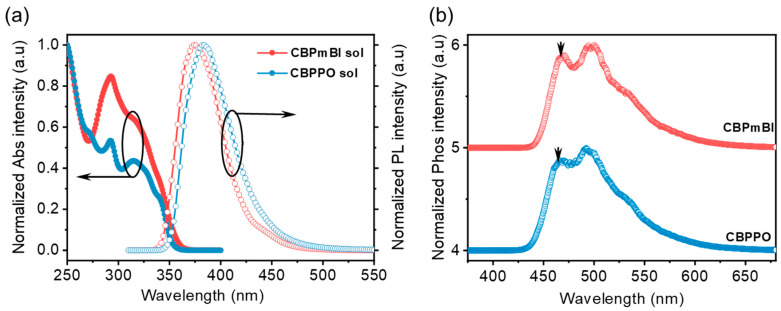
(**a**) UV-Vis absorption and photoluminescence (PL) spectra of **CBPPO** and **CBPmBI** measured in dilute dichloromethane solutions under ambient temperature. (**b**) Phosphorescence spectra of the two compounds (**CBPPO** and **CBPmBI**) recorded in 2-methyltetrahydrofuran glass matrices at 77 K.

Combined with Figure 2’s spectral curves and Table 1’s performance parameters, **CBPPO** and **CBPmBI** exhibit distinct photophysical properties, with the structural difference in their EWGs being the core cause. Both compounds show characteristic absorption peaks at ~292 nm and ~315 nm, originating from carbazole-centered π-π* transitions—consistent with typical optical behavior of carbazole-based materials [31]. Their optical energy gaps (E_g_) were calculated via solution-state absorption onset (to avoid molecular aggregation interference): **CBPPO** has an E_g_ of 3.50 eV, while **CBPmBI**’s E_g_ is slightly smaller (3.40 eV).

In CH_2_Cl_2_ solution, their photoluminescence (PL) emission peaks differ: **CBPPO**’s is at ~375 nm, while **CBPmBI**’s is red-shifted to ~380 nm. This red shift aligns with **CBPmBI**’s smaller E_g_, conforming to the “smaller E_g_ → longer emission wavelength” rule in organic semiconductors.

The value of E_T_ was determined via 77 K phosphorescence spectra (2-methyltetrahydrofuran as glass matrix). Extracting the highest-energy vibronic sub-band energy (Figure 2b) revealed both derivatives have the same E_T_ (2.67 eV)—notably higher than FIrpic’s E_T_ (2.65 eV) [31]. This meets the core requirement for blue phosphorescent OLED hosts (host E_T_ > guest E_T_ to suppress reverse exciton transfer), confirming **CBPPO** and **CBPmBI**’s potential as hosts for FIrpic-based blue PhOLEDs.

### 2.4. Electrochemical Properties

**Figure 3:**
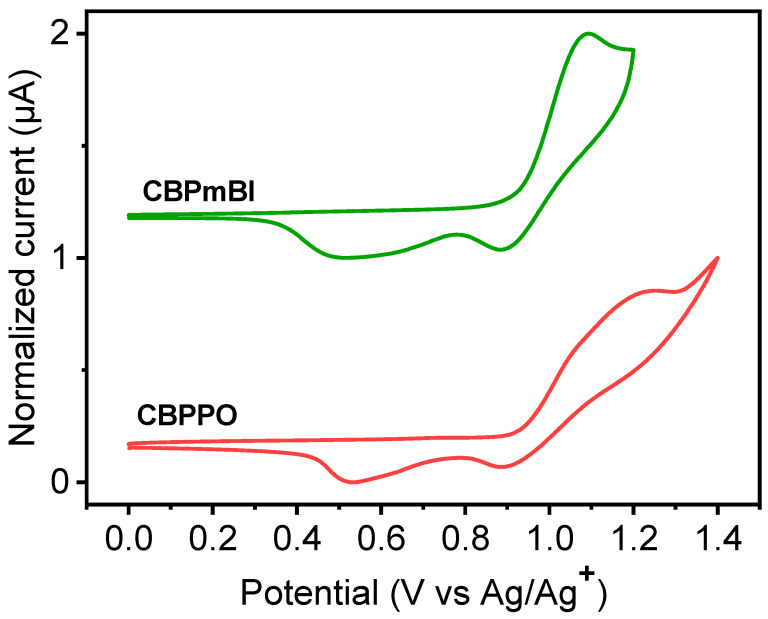
Cyclic voltammograms for the target compounds in dichloromethane (CH_2_Cl_2_) solution corresponding to the oxidation process.

The oxidative electrochemical behaviors of **CBPPO** and **CBPmBI** were characterized via cyclic voltammetry (CV; see Figure 3). Both host materials exhibited distinct oxidation waves within the electrochemical window of CH_2_Cl_2_. During the oxidation scan, an additional peak appeared at approximately 0.6 V for both compounds, which is most likely attributed to the electrochemical activity of the C3 and C6 positions on the carbazole moiety [34].

Affected by their electron-withdrawing groups (similar in structure but with slight differences), **CBPPO** and **CBPmBI** showed oxidation potentials of 1.05 V and 0.95 V, respectively. The highest occupied molecular orbital (HOMO) and lowest unoccupied molecular orbital (LUMO) energy levels of these compounds were calculated using their oxidation onset potentials and absorption spectra. Specifically, the HOMO levels of **CBPPO** and **CBPmBI** were −5.70 eV and −5.60 eV, respectively. Their corresponding LUMO levels—derived from the HOMO values and optical energy gaps (3.50 eV for **CBPPO**, 3.40 eV for **CBPmBI**)—were nearly identical at approximately −2.20 eV. Moreover, the HOMO levels of both derivatives were significantly higher than that of the traditional **CBP** host (−6.0 eV) [35], indicating a lower hole-injection barrier from the hole-transporting layer to the emissive layer compared with **CBP**.

### 2.5. Theoretical Calculations

**Figure 4:**
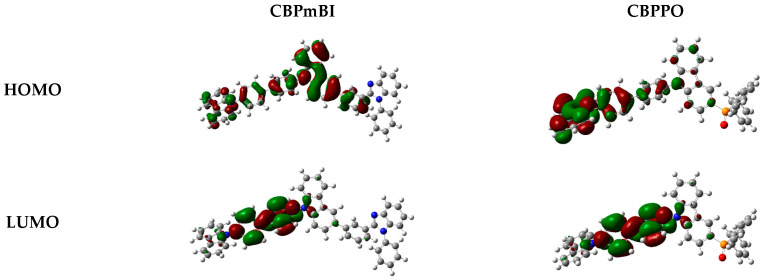
Calculated spatial distributions of the HOMO and LUMO energy densities of **CBPPO** and **CBPmBI**.

Figure 4 illustrates the electron density distributions of both HOMO and LUMO in the two target compounds, which were derived via quantum chemical calculations. Both **CBPPO** and **CBPmBI** exhibit significant overlap between their HOMO and LUMO electron distributions, though their orbital localization patterns differ distinctly.

For the **CBPmBI** molecule, its HOMO is predominantly concentrated on the two electron-rich carbazole units and the linking biphenyl bridge—regions that possess high electron density owing to the electron-donating characteristic of carbazole. In contrast, its LUMO is confined to the benzene ring that bridges the two carbazole units, a distribution driven by the electron-withdrawing effect of the benzimidazole group. As for **CBPPO**, its HOMO shows a more restricted distribution, mainly localized on the carbazole moiety distant from the diphenylphosphine oxide group; this is likely because the electron-withdrawing diphenylphosphine oxide reduces electron density in the adjacent carbazole. Meanwhile, **CBPPO**’s LUMO is spread across two benzene moieties, which correlates with the electron-accepting property of the diphenylphosphine oxide group. Notably, the calculated HOMO/LUMO energy levels for both compounds are identical, at −5.4 eV/−2.2 eV. This result is in good consistency with the experimental data summarized in Table 1, validating the reliability of the quantum chemical calculation method used in this study.

### 2.6. Electroluminescence Performance

**Figure 5:**
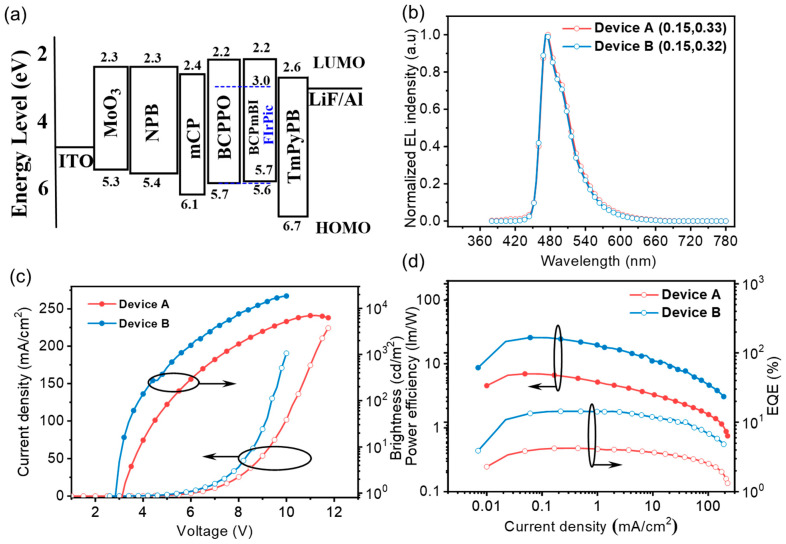
(**a**) Energy level diagrams of the materials employed in Device A and Device B, (**b**) Electroluminescence (EL) spectra of the blue PhOLEDs employing distinct host materials, (**c**) Current density-voltage-luminance (J-V-L) characteristics, and (**d**) External quantum efficiency (EQE) and power efficiency as a function of current density.

To assess the applicability of **CBPPO** and **CBPmBI** as bipolar host materials for blue PhOLEDs, devices doped with FIrpic were fabricated adopting a simplified device structure: Indium tin oxide (ITO)/Molybdenum trioxide (MoO_3_) (10 nm)/1,4-bis[(1-naphthyl phenyl)amino]biphenyl (NPB) (40 nm)/N,N′-dicarbazolyl-3,5- benzene (mCP) (5 nm)/Host:6 wt% FIrpic (20 nm)/3,3′-(5′-(3-(pyridine-3-yl)phenyl)-[1,1′:3′,1″-terphenyl]-3,3″-diyl)dipyridine (TmPyPB) (40 nm)/Lithium fluoride (LiF) (1 nm)/Al (150 nm) (The specific structures are shown in Figure S5). Device A used **CBPmBI** as the host, while Device B adopted **CBPPO**—each layer was designed for specific functions: MoO_3_ and LiF served as hole- and electron-injecting layers to reduce carrier injection barriers; NPB acted as the hole-transporting layer to guide holes toward the emissive layer; mCP functioned as an exciton-blocking layer to prevent exciton diffusion to adjacent layers (avoiding non-radiative loss); TmPyPB played dual roles (electron-transporting and hole-blocking) to balance carrier influx into the emissive layer; the emissive layer itself combined host and 6 wt% FIrpic—optimization confirmed this doping concentration yielded the best electroluminescence (EL) performance for both hosts. The schematic energy level diagrams of the two devices are presented in Figure 5a.

Device performance characteristics (current density-brightness-voltage, efficiency-brightness) are shown in Figure 5, with key parameters summarized in Table 2. Device B (**CBPPO**-based) stood out: it had a low turn-on voltage of 2.9 V, and its maximum external quantum efficiency (η_EQE,max_ = 14.4%), current efficiency (η_c,max_ = 28.0 cd/A), and power efficiency (η_p,max_ = 24.4 lm/W) were comparable to the highest reported values for FIrpic-based blue PhOLEDs. More notably, it exhibited negligible efficiency roll-off—at practical high brightness (1000 cd/m^2^ for indoor displays, 5000 cd/m^2^ for outdoor use), its EQE remained 14.2% and 14.1%, respectively. This stability arises from **CBPPO’s** balanced bipolar transport: it efficiently injects/transports both electrons and holes, minimizing triplet-triplet annihilation and triplet-polaron quenching (major causes of roll-off).

Device A (**CBPmBI**-based) also performed well with a low turn-on voltage of 3.1 V (at 1 cd/m^2^), but its efficiencies were lower (η_c,max_ = 8.5 cd/A, η_p,max_ = 7.0 lm/W, η_EQE,max_ = 4.2%), and its maximum EQE was only ~25% of Device B′s. This gap stems from two factors: firstly, while **CBPmBI**’s triplet energy (2.67 eV) is sufficient for FIrpic, its slightly weaker electron-withdrawing group may cause minor exciton leakage to the host; secondly, the electron injection barrier between **CBPmBI**’s LUMO (−2.20 eV) and TmPyPB′s LUMO (evident in Figure 5a) is larger than **CBPPO**’s, leading to unbalanced charge recombination in the emissive layer.

The low turn-on voltages of both devices (2.9–3.0 V) are attributed to their hosts’ favorable energy levels and bipolar properties: their HOMO levels (−5.70 eV for **CBPPO**, −5.60 eV for **CBPmBI**) align well with NPB’s HOMO (−5.4 eV) to lower hole-injection barriers, while their LUMO (−2.20 eV) matches TmPyPB′s to facilitate electron injection—collectively enabling efficient carrier transport at low voltages.

Figure 5b presents the electroluminescence (EL) spectra of OLED devices employing **CBPPO** and **CBPmBI** as host materials, respectively. Notably, the two devices exhibit nearly overlapping EL profiles, with their emission signals originating exclusively from the FIrpic guest emitter rather than the host materials. Despite the minimal E_T_ disparity between **CBPPO/CBPmBI** (2.67 eV) and FIrpic (2.65 eV), no detectable emission from the **CBPPO** or **CBPmBI** hosts was observed in the EL spectra, indicating effective confinement of excitons on the FIrpic emitter. This phenomenon verifies that the slight E_T_ difference between the hosts and FIrpic is sufficient to suppress reverse exciton transfer and host-related luminescence leakage. On the contrary, leveraging these hosts yielded more favorable device performance than traditional high-triplet-energy hosts [36,37], as evidenced by reduced driving voltage and enhanced maximum brightness of the fabricated OLEDs.

## 3. Conclusions

Two bipolar host materials (**CBPPO**, **CBPmBI**) were successfully synthesized via efficient routes (Ni (II)/Zn-catalyzed cross-coupling for **CBPPO**, Suzuki coupling for **CBPmBI**). Introducing EWGs to the CBP backbone significantly enhanced their performance: both exhibited high thermal stability (T_g_ = 147–157 °C, T_d_ = 460–494 °C) and triplet energy (E_T_ = 2.67 eV), resolving the inherent limitations of pristine CBP. Among them, **CBPPO**-based blue PhOLEDs showed favorable electroluminescence performance: maximum external quantum efficiency (EQE_max_) reached 14.4%, with negligible efficiency roll-off—maintaining 14.1% EQE even at 5000 cd/m^2^. This is attributed to **CBPPO**’s balanced bipolar charge transport, which suppresses exciton loss. This work demonstrates that EWG modification of CBP is a facile, effective strategy to tune host material properties. It provides a viable route for designing high-performance bipolar hosts, advancing the development of efficient blue PhOLEDs.

## Figures and Tables

**Table 1:** Compilation of Physical Property Measurements for **CBPPO** and **CBPmBI**.

Host	λ_max,abs_ (nm) *^a^*	λ_max,PL_ (nm) *^a^*	E_g_ (eV) *^b^*	HOMO/LUMO (eV) *^c^*		HOMO/LUMO (eV) *^d^*		E_T_ (eV) *^e^*	T_g_/T_d_ *^f^* (°C)
**CBPmBI**	316/292	383	3.4	−5.6	−2.2	−5.4	−2.4	−2.67	157/494
**CBPPO**	315/292	375	3.5	−5.7	−2.2	−5.4	−2.4	−2.67	147/460

*^a^* Measured in dichloromethane solution at ambient temperature. *^b^* Derived from the absorption onset of dichloromethane solutions. *^c^* Evaluated from cyclic voltammetry (CV) data; HOMO = −E_1/2_^ox^ (vs. Ag/Ag^+^) − EAg/Ag^+^, where the Ag/Ag^+^ energy level relative to the vacuum level is 4.65 eV. *^d^* Obtained via density functional theory (DFT) calculations. *^e^* Measured in 2-MeTHF glass matrices at 77 K. *^f^ T*_g_ and *T*_d_ denote the glass transition temperature and decomposition temperature, respectively.

**Table 2:** EL data of devices A and B.

Device	Host	V_on_ (v) *^a^*	L_max_ [cd/m^2^] (V at L_max_, V_max_) *^a^*	η_c_ *^b^* [cd/A]	η_p_ *^c^* [lm/W]	η_EQE_ *^d^* [%]	CIE [x, y] *^e^*
A	CBPmBI	3.1	6994 (11.3)	8.5	7.0	4.2	(0.15, 0.33)
B	CBPPO	2.9	18,600 (10.0)	28.0	25.8	14.4	(0.14, 0.32)

*^a^* V_on_: voltage corresponding to a brightness of 1 cd/m^2^, V_max_: voltage at the maximum luminance; *^b^* Peak current efficiency; *^c^* Peak power efficiency; *^d^* Peak external quantum efficiency; *^e^* Determined at the brightness of 100 cd/m^2^.

## Data Availability

The original contributions presented in this study are included in the article/Supplementary Materials. Further inquiries can be directed to the corresponding authors.

## Supplementary Materials

The following supporting information can be downloaded at: https://www.mdpi.com/article/10.3390/molecules31010012/s1, Figure S1. ^1^H NMR spectra (400 MHz, CDCl_3_, 298 K) of compound CBPPO; Figure S2. ^13^C NMR spectra (100 MHz, CDCl_3_, 298 K) of compound CBPPO; Figure S3. ^1^H NMR spectra (400 MHz, CDCl_3_, 298 K) of compound CBPmBI; Figure S4. ^13^C NMR spectra (100 MHz, CDCl_3_, 298 K) of compound CBPmBI; Figure S5. Chemical structures of NPB, mCP, FIrpic, and TmPyPB.
