# Supplementary material for: Templated Bipolar Host Materials for Blue Phosphorescent Organic Light-Emitting Devices with Negligible Efficiency Roll-Offs
Source: Molecules. 2025 Dec 19;31(1):12. doi: 10.3390/molecules31010012 (PMC12786962; doi:10.3390/molecules31010012)
Supplement: Supplementary file 1 [file molecules-31-00012-s001.zip › molecules-4021624-supplementary.pdf]

Supporting information for

Templated Bipolar Host Materials for Blue Phosphorescent Organic Light-Emitting Devices with Negligible Efficiency Roll-offs

Hong Huang[1], Tao Hua[1], Nengquan Li[1], Youming Zhang[1,*], Manli Huang[1], Xiaolu Zhou[1], Shaoqing Zhuang[2,*], Guohua Xie[3,*]

1. Institute of Technology for Future Industry, School of Science and Technology Instrument Application Engineering, Shenzhen University of Information Technology, Shenzhen 518172, P. R. China;

2. Wuhan Sunshine Optoelectronics Tech Co., Ltd, New Energy Building, No. 999 Gaoxin Avenue, Wuhan, 430074, P. R. China.

3. Institute of Flexible Electronics (IFE, Future Technologies), Institute of Future Display Technology, Tan Kah Kee Innovation Laboratory, Xiamen University, Xiamen 361102, China

E-mail: zhangym@sziit.edu.cn (Y.Z.); zhuangshaoqing@sunshine-oled.com (S.Z.); ifeghxie@xmu.edu.cn (G.X.).

Materials and measurements

Reagent Handling and Reaction Conditions

All chemical reagents were purchased from Aldrich and utilized directly without further purification unless specifically noted otherwise. To eliminate interference from moisture and oxygen, all synthetic reactions were conducted under an atmosphere of dry nitrogen (N_2), ensuring the stability of sensitive intermediates and products throughout the reaction process.

^{1}H and ^{13}C Nuclear Magnetic Resonance (NMR) Spectroscopy

Nuclear magnetic resonance measurements were carried out on a Bruker-AF301 AT spectrometer to confirm the molecular structure of the target compounds and intermediates. Specifically, ^{1}H NMR spectra were recorded at a resonance frequency of 400 MHz, while ^{13}C NMR spectra were obtained at 100 MHz. Deuterated chloroform ($CDCl_3$) was employed as the test solvent for all samples. Chemical shifts (δ) were referenced to the characteristic peaks of $CDCl_3$: 7.26 ppm was used as the internal standard for ^{1}H NMR, and 77.16 ppm served as the reference for ^{13}C NMR, ensuring the accuracy of structural assignment.

Atmospheric Pressure Chemical Ionization Mass Spectrometry (APCI-MS)

To further verify the molecular weight and purity of the synthesized compounds, APCI-MS analysis was performed on an Agilent 1100 LC/MSD Trap mass spectrometer. This technique enabled the detection of the molecular ion peaks of CBPPO and CBPmBI, providing direct evidence for the successful synthesis of the target products.

UV-Vis Absorption and Photoluminescence (PL) Spectroscopy

Optical properties of the compounds were characterized via two complementary spectroscopic techniques:

1. UV-Vis absorption spectra: Measured using a Shimadzu UV-3600 spectrophotometer in dilute dichloromethane (CH_2Cl_2) solution (concentration = 10^{-5} M) at room temperature (298 K). This test quantified the characteristic absorption bands

of the carbazole-based moieties and determined the optical energy gap (E_g) from the absorption onset.

2. Photoluminescence (PL) spectra: Acquired with an Edinburgh FLSP920 fluorescence spectrometer under the same solution and temperature conditions as the UV-Vis tests. The PL spectra reflected the intrinsic emission behavior of the host materials in the solution state, with emission peaks used to analyze the influence of electron-withdrawing groups on the optical properties.

Low-Temperature Phosphorescence Spectroscopy

Triplet energy (E_T) values of CBPPO and CBPmBI were determined via phosphorescence spectroscopy at 77 K, using 2-methyltetrahydrofuran (2-MeTHF) as the glass matrix. This low-temperature environment suppressed non-radiative transitions, allowing the capture of the high-energy vibronic sub-band of the phosphorescence spectrum, which was then used to calculate the ET of the materials (critical for evaluating their suitability as blue PhOLED hosts).

Differential Scanning Calorimetry (DSC)

Thermal transition properties of the compounds were evaluated via DSC analysis on a PerkinElmer DSC 2920 instrument. The test was conducted under a nitrogen atmosphere with a heating rate of 10 °C/min, over a temperature range of 30–300 °C. To eliminate the interference of residual thermal history from sample preparation, the glass transition temperature (Tg) was extracted from the second heating scan, ensuring the accuracy of the thermal transition data.

Thermogravimetric Analysis (TGA)

Thermal stability was assessed using TGA on a PerkinElmer Pyris1 thermogravimetric analyzer. The samples were heated from 30 °C to 700 °C at a rate of 10 °C/min under nitrogen protection. The decomposition temperature (T_d) was defined as the temperature corresponding to a 5% weight loss of the sample, a key indicator of the material's ability to withstand OLED fabrication thermal stress.

Cyclic Voltammetry (CV)

Electrochemical oxidation behaviors and frontier molecular orbital energy levels were measured via CV on an EG&G 283 Potentiostat using a standard three-electrode

system:

- Working electrode: Platinum (Pt) disk electrode
- Counter electrode: Platinum (Pt) wire electrode
- Reference electrode: Ag/$AgNO_3$ electrode (0.1 M $AgNO_3$ in acetonitrile)

The electrolyte was 0.1 M tetrabutylammonium hexafluorophosphate (Bu_4NPF_6) dissolved in CH_2Cl_2, and the solution was purged with nitrogen for 10 min before testing to remove dissolved oxygen. The highest occupied molecular orbital (HOMO) energy level was calculated using the formula: HOMO = -($E_{ox\ onset}$ + 4.65) eV (where 4.65 eV is the vacuum level of the Ag/$AgNO_3$ reference electrode). The lowest unoccupied molecular orbital (LUMO) energy level was derived from the HOMO value and the optical E_g (obtained from UV-Vis absorption onset) via the equation: LUMO = HOMO - E_g.

Computational Details

The geometrical configurations and electronic characteristics of the target compounds were investigated using the Amsterdam Density Functional (ADF) 2009.01 program package. For structural optimization, the B3LYP hybrid functional—incorporating the Becke three-parameter exchange functional and Lee-Yang-Perdew correlation functional[16]—was employed in conjunction with the 6-31G(d) atomic basis set. Subsequently, the electronic structures of the optimized geometries were computed at the τ-HCTHhyb/6-311^{++}G(d,p) level of theory[17]. Visualization of the molecular orbitals was carried out via the ADFview software module.

Device Fabrication and Characterization

The materials used for device fabrication included commercially sourced components: molybdenum trioxide (MoO_3, as the hole-injection material), 1,4-bis [(1-naphthylphenyl)amino]biphenyl (denoted as NPB, as the hole-transporting material), N,N'-dicarbazolyl-3,5-benzene (denoted as mCP, as the electron/exciton-blocking material), and 3,3'-(5'-(3-(pyridine-3-yl)phenyl)-[1,1':3',1''-terphenyl]-3,3''-diyl) dipyridine (denoted as TmPyPB, as the electron-transporting material).

Indium tin oxide (ITO)-coated glass substrates were adopted, which were commercially available and featured a sheet resistance of 20 Ω per square. Prior to device fabrication, the ITO glass substrates underwent thorough pre-cleaning

procedures, followed by an oxygen plasma treatment for 2 minutes to optimize surface properties.

Subsequently, thin-film deposition was conducted in a vacuum chamber: MoO_3 was first deposited onto the treated ITO substrate, followed by the sequential deposition of NPB, mCP, the emissive layer, and TmPyPB. Finally, a cathode structure consisting of lithium fluoride (LiF) and aluminum (Al) was deposited sequentially on the top layer, with the entire deposition process carried out under a vacuum of 10^{-6} Torr.

The current density-voltage-brightness (J-V-L) characteristics of the fabricated devices were measured using a Keithley 2400 Source Meter, which was paired with a calibrated silicon photodiode to capture brightness data. Electroluminescence (EL) spectra of the devices were recorded using a PR655 spectrometer. External quantum efficiency (EQE) values were computed in accordance with the methods reported in the previous literature[18].

Synthesis of Materials

N,N'-dicarbazolyl-4,4'-biphenyl (CBP) was synthesized following the protocol described in the existing literature[19]. All coupling reactions involved in the preparation of target derivatives were carried out under a dry nitrogen atmosphere, and strict measures were taken to avoid light exposure throughout the reaction process to prevent potential side reactions that might affect product purity.

*Synthesis of 9-(4'-(9H-carbazol-9-yl)-[1,1'-biphenyl]-4-yl)-3-bromo-9H-carbazole (**1**)*

To synthesize compound **1**, a solution of N,N'-dicarbazolyl-4,4'-biphenyl (CBP, 5.0 g, 10.3 mmol) and anhydrous silica gel (100 g) in chloroform ($CHCl_3$, 250 mL) was first prepared. Newly recrystallized N-bromosuccinimide (NBS, 2.2 g, 12.4 mmol) was then added to this solution, and the resulting mixture was stirred at room temperature for approximately 48 hours. After the reaction finished, the mixture was filtered to remove insoluble residues, and the collected filtrate was further purified via recrystallization. Finally, the target product was obtained as a white powder with a yield of 95%. MS (APCI): calcd for $C_{36}H_{23}N_2Br$: 526.1, found, 527.1 $(M+1)^+$.

*Synthesis of (9-(4'-(9H-carbazol-9-yl)-[1,1'-biphenyl]-4-yl)-9H-carbazol-3-yl) diphenylphosphine oxide (**CBPPO**)*

Into a 20.0 mL N,N-dimethylacetamide (DMAc) solution containing a mixture of

nickel(II) chloride hexahydrate ($NiCl_2·6H_2O$, 0.07 g, 0.3 mmol), zinc powder (0.39 g, 6.0 mmol), 2,2′-bipyridine (bpy, 0.09 g, 0.6 mmol), and compound **1** (0.53 g, 1.0 mmol), biphenylphosphine oxide (0.4 g, 2.0 mmol) was added. The resulting reaction mixture was stirred continuously at 110 °C for 24 hours. Once the reaction was completed, the mixture was cooled down to room temperature and then treated with dichloromethane (CH_2Cl_2) and deionized water. The organic phase was separated from the aqueous phase, and then dried over anhydrous magnesium sulfate ($MgSO_4$) to remove residual moisture. Subsequently, the crude product obtained after removing the drying agent and evaporating the solvent was purified by silica gel column chromatography. A mixed solvent of dichloromethane and methanol was used as the eluent, and this purification process yielded the target cross-coupled product. The overall yield of the product was 54%. ^{1}H-NMR (400 MHz, $CDCl_3$): δ (ppm) 8.61 ~ 8.58 (d, J = 12.4 Hz, 1H), 8.18 ~ 8.12 (m, 3H), 7.92 ~ 7.89 (m, 4H), 7.78 ~ 7.63 (m, 9H), 7.57 ~ 7.42 (m, 13H), 7.36 ~ 7.30 (m, 3H)。 ^{13}C-NMR (100 MHz, $CDCl_3$): δ (ppm) 142.72, 141.40, 140.78, 139.96, 139.03, 137.43, 136.45, 133.91, 132.87, 132.27, 132.17, 131.82, 131.80, 129.48, 129.36, 128.67, 128.54, 128.42, 127.51, 126.90, 126.03, 125.44, 125.33, 123.62, 123.52, 123.49, 123.15, 123.00, 122.06, 121.00, 120.80, 120.39, 120.12, 110.18, 110.02, 109.88, 109.79。 MS (APCI): calcd for $C_{48}H_{33}N_2OP$: 684.2, found, 685.4 $(M+1)^+$。

Synthesis of 9-(4'-(9H-carbazol-9-yl)-[1,1'-biphenyl]-4-yl)-3-(3-(1-phenyl-1H-benzo [d]imidazol-2-yl)phenyl)-9H-carbazole ***(CBPmBI)***

To synthesize the final compound **CBPmBI**, a reaction mixture was first prepared by combining compound 1 (0.53 g, 1.0 mmol), 1-phenyl-2-(3-(4,4,5,5- tetramethyl-1,3,2-dioxaborolan-2-yl)phenyl)-1H-benzo[d]imidazole (0.48 g, 1.2 mmol), tetrakis(triphenylphosphine)palladium ($Pd(PPh_3)_4$, 115.5 mg, 0.1 mmol), a 2.0 M aqueous solution of potassium carbonate (K_2CO_3, 5.0 mL, 10.0 mmol), toluene (50 mL), and ethanol (25.0 mL). This mixture was heated and stirred continuously at 100 °C for 24 hours. Once the reaction concluded, the mixture was cooled to room temperature, and dichloromethane (CH_2Cl_2) was added to the reaction system to extract the product. The organic phase was then separated from the aqueous phase, washed with saturated brine to remove water-soluble impurities, and subsequently dried using anhydrous

magnesium sulfate ($MgSO_4$) to eliminate residual moisture. After filtering out the drying agent, the solvent in the organic phase was evaporated under reduced pressure, leaving a solid residue. This residue was further purified via silica gel column chromatography, where a mixed eluent of ethyl acetate and chloroform (volume ratio, v/v = 1:1) was used. Through this purification process, the target compound was obtained as a white solid with a reaction yield of 88%. ^{1}H-NMR (400 MHz, $CDCl_3$): δ (ppm) 8.20 ~ 8.17 (m, 3H), 8.10 (s, 1H), 7.96 ~ 7.90 (m, 6H), 7.73 ~ 7.71 (d, *J* = 8.0 Hz, 5H), 7.66 ~ 7.60 (m, 4H), 7.53 ~ 7.43 (m, 11H), 7.38 ~ 7.32 (m, 6H)。 ^{13}C-NMR (100 MHz, $CDCl_3$): δ (ppm) 152.40, 141.81, 141.29, 140.82, 140.40, 139.43, 139.23, 137.32, 137.25, 137.11, 132.69, 130.20, 130.05, 128.90, 128.64, 128.58, 128.52, 128.41, 128.33, 127.71, 127.51, 127.43, 126.30, 126.03, 125.42, 124.01, 123.58, 123.52, 123.46, 123.11, 120.44, 120.39, 120.33, 120.10, 119.88, 118.92, 110.49, 110.03, 109.82。 MS (APCI): calcd for $C_{55}H_{36}N_4$: 752.3, found, 753.6 $(M+1)^+$。

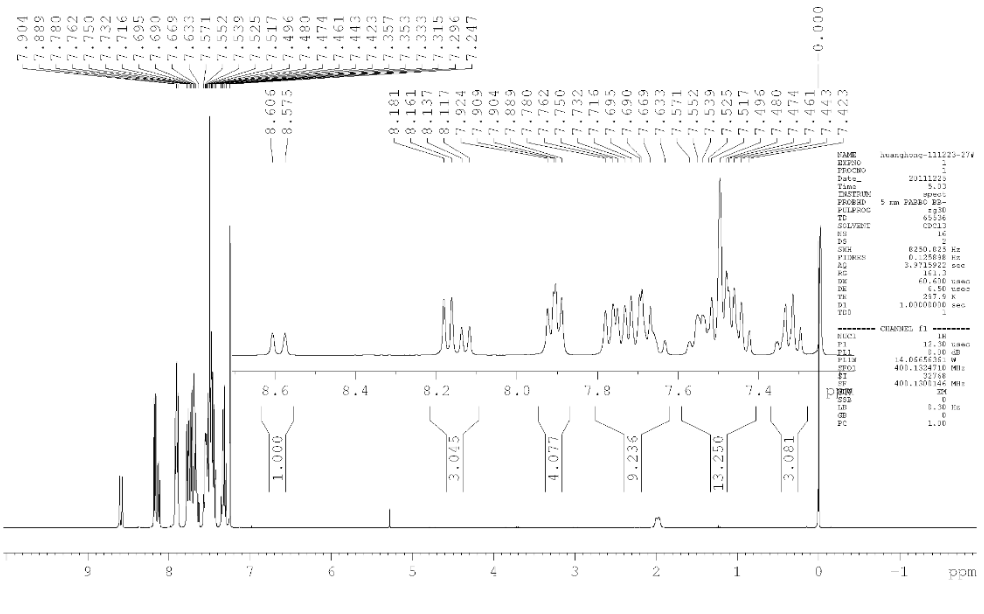

Figure S1. ^{1}H NMR spectra (400 MHz, $CDCl_3$, 298 K) of compound **CBPPO**.

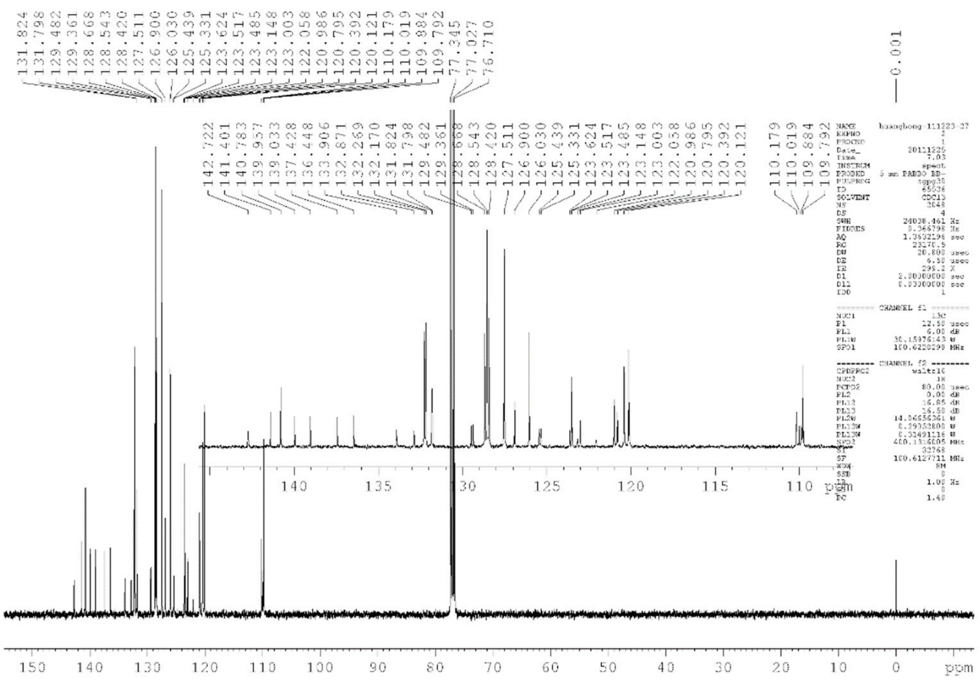

Figure S2. ^{13}C NMR spectra (100 MHz, $CDCl_3$, 298 K) of compound **CBPPO**.

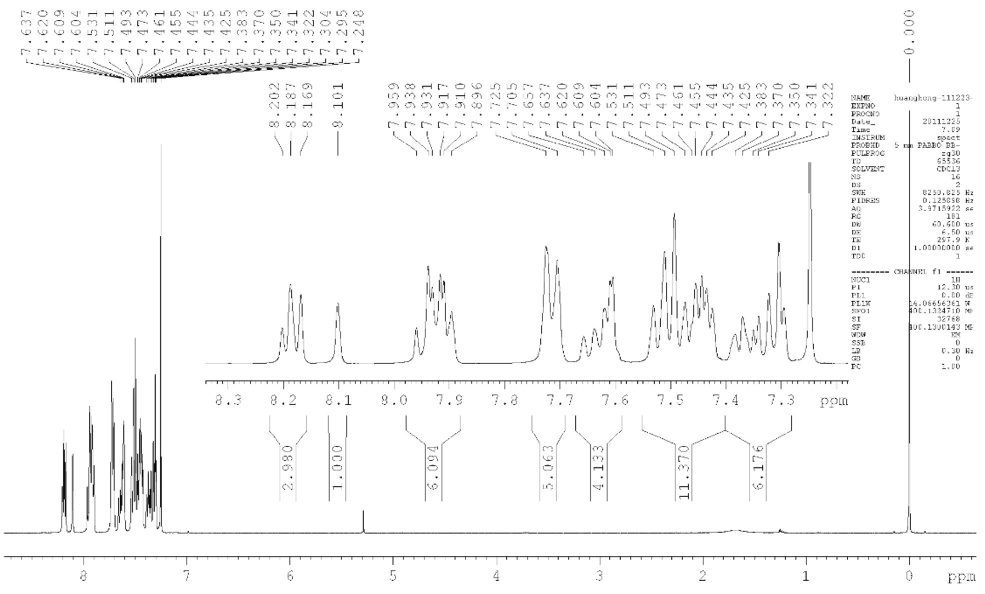

Figure S3. ^{1}H NMR spectra (400 MHz, $CDCl_3$, 298 K) of compound **CBPmBI**.

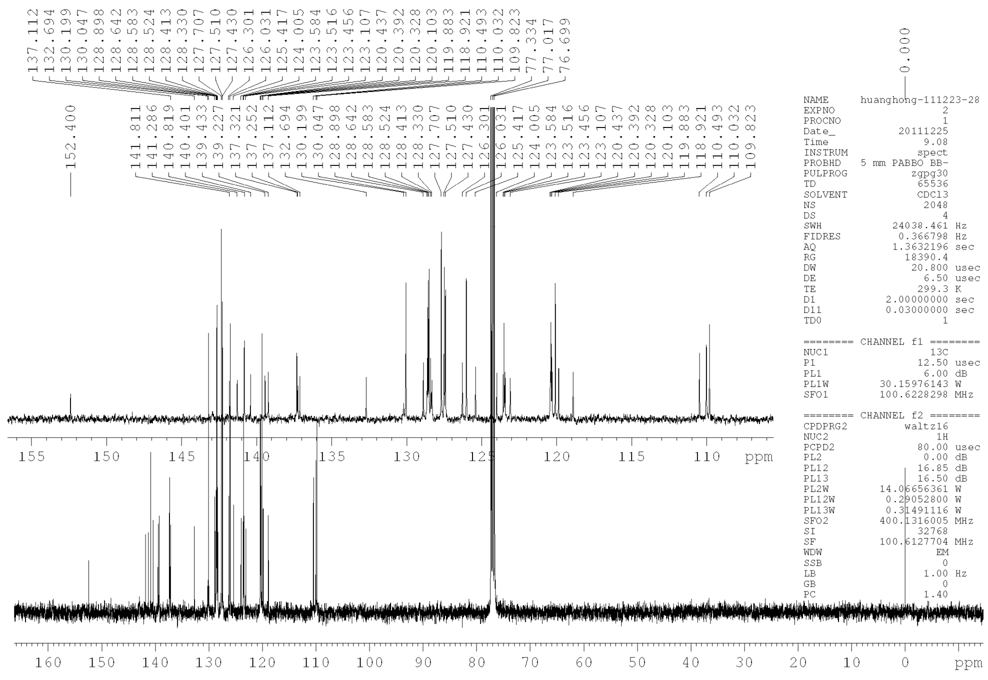

Figure S4. ^{13}C NMR spectra (100 MHz, $CDCl_3$, 298 K) of compound **CBPmBI**.

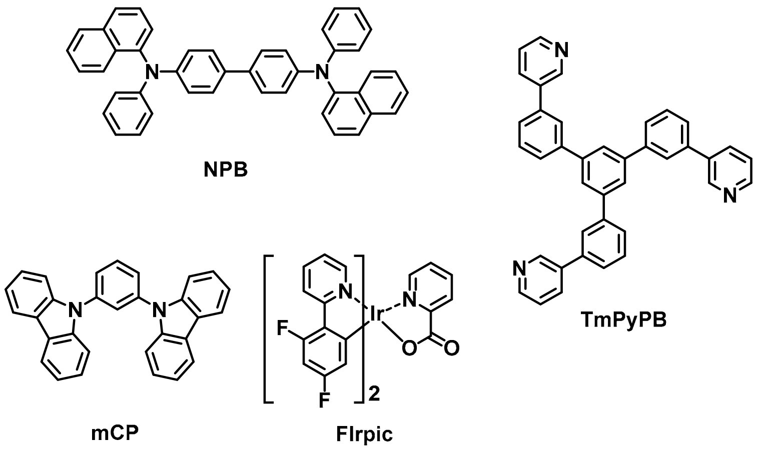

Figure S5. Chemical structures of NPB, mCP, FIrpic, and TmPyPB.
